# Incidence and predictors of acute respiratory distress syndrome in sepsis: a systematic review and meta-analysis

**DOI:** 10.3389/fmed.2026.1695735

**Published:** 2026-01-23

**Authors:** Wenlong Li, Minxi Liao, Cheng Huang, Changjie Mei, Jiaqian Deng

**Affiliations:** 1 Emergency Department, Ganzhou People’s Hospital, Ganzhou, Jiangxi, China; 2 School of Chinese Medicine, Hong Kong Baptist University, Kowloon Tong, Hong Kong SAR, China; 3 Cardiology Department, Ganzhou People’s Hospital, Ganzhou, Jiangxi, China

**Keywords:** acute respiratory distress syndrome, incidence, meta-analysis, risk factors, sepsis, sequential organ failure assessment score

## Abstract

**Background:**

Acute respiratory distress syndrome (ARDS) is a serious and frequent complication of sepsis, carrying a high risk of both morbidity and mortality. Despite extensive research on ARDS in patients with sepsis, the reported incidence and associated risk factors remain inconsistent, with findings varying considerably across clinical settings.

**Objective:**

To systematically evaluate the pooled proportion (cumulative incidence) of ARDS among adult patients with sepsis and to identify independent risk factors associated with its development.

**Methods:**

A comprehensive search of PubMed, Embase, Cochrane Library, Web of Science, and Scopus was performed for studies published up to May 30, 2025. Observational studies reporting ARDS incidence or risk factors in adult septic populations were included. Data extraction and quality assessment were performed independently by two reviewers to minimize bias. Pooled estimates were generated using a random-effects model, while heterogeneity across studies was quantified with the *I*^2^ statistic. Potential publication bias was examined using both Egger’s and Begg’s tests.

**Results:**

A total of 24 studies involving 23,394 septic patients were included. The pooled incidence of ARDS among septic patients was 34.0% (95% CI: 29.0–39.3%), with considerable heterogeneity (*I*^2^ = 96.5%). Significant risk factors for ARDS included pneumonia (OR = 2.88, 95% CI: 2.07–3.99), pancreatitis (OR = 2.45, 95% CI: 1.87–3.21), septic shock (OR = 1.78, 95% CI: 1.38–2.31), smoking (OR = 2.23, 95% CI: 1.33–3.75), elevated Sequential Organ Failure Assessment (SOFA) (OR per point = 1.15, 95% CI: 1.10–1.21) and APACHE II scores (OR per point = 1.12, 95% CI: 1.04–1.20), CRP (OR = 1.01), and serum creatinine (Scr) (OR = 1.07). No significant association was found for age, gender, diabetes, or cirrhosis.

**Conclusion:**

ARDS complicates approximately one-third of sepsis cases, with its development strongly associated with infection site, disease severity, and systemic inflammation. Routine evaluation of clinical and biochemical markers, including SOFA and Acute Physiology and Chronic Health Evaluation II (APACHE II) scores, pneumonia, and C-reactive protein (CRP) levels, may aid in early risk stratification. These findings support the need for targeted monitoring and prevention strategies in high-risk septic patients.

**Systematic review registration:**

https://www.crd.york.ac.uk/PROSPERO, (CRD420251118623).

## Background

Acute respiratory distress syndrome (ADRS) is a severe, life-threatening form of acute respiratory failure characterized by diffuse alveolar damage, non-cardiogenic pulmonary edema, and severe hypoxemia (1, 2). Despite advances in supportive care, ARDS continues to be associated with high mortality, ranging from 30% to over 40% depending on disease severity and underlying conditions (3, 4). One of the leading precipitating factors for ARDS is sepsis, a dysregulated host response to infection that results in life-threatening organ dysfunction (5).

Sepsis is globally prevalent, affecting an estimated 48.9 million individuals annually and contributing to 11 million deaths (6). Among these cases, ARDS develops in up to 6–33% of septic patients, depending on population characteristics and diagnostic criteria applied (7–9). The Berlin definition of ARDS, introduced in 2012, has improved diagnostic standardization, yet heterogeneity in diagnostic practice and clinical populations continues to challenge epidemiological estimations (10, 11).

The onset of ARDS in patients with sepsis is shaped by multiple factors, arising from the interaction between host characteristics, infectious agents, and environmental influences. Numerous investigations have attempted to determine independent predictors of ARDS in this context, but the findings have been inconsistent. Among clinical indicators, severity scores such as the Acute Physiology and Chronic Health Evaluation II (APACHE II) and the Sequential Organ Failure Assessment (SOFA) have shown a consistent association with elevated risk of ARDS (12–14). Inflammatory biomarkers such as C-reactive protein (CRP) and elevated lactate have also been implicated as possible indicators of progression to ARDS.

Site of infection has emerged as a relevant clinical factor, with pulmonary infections being strongly associated with ARDS development (15, 16). Conversely, non-pulmonary infections such as abdominal or soft tissue sources may confer a different risk profile (17, 18). Comorbidities such as chronic obstructive pulmonary disease (COPD), smoking history, and diabetes mellitus have also been variably associated with ARDS susceptibility, though findings remain inconsistent across studies (19, 20).

Moreover, the role of therapeutic interventions for sepsis—such as corticosteroid use—remains controversial. While steroids may modulate the inflammatory cascade, their impact on ARDS incidence in sepsis patients is unclear, with some studies suggesting a neutral or even protective effect, while others raise concerns over immunosuppression and delayed pathogen clearance (21, 22).

Although numerous cohort and case–control studies have addressed these questions individually, the reported results are often conflicting due to variations in study design, population characteristics, and sample sizes. Recent meta-analyses have attempted to synthesize this data, but have either focused on broader ARDS populations or lacked updated inclusion of emerging studies from diverse regions (3).

Given the clinical importance of early recognition and targeted prevention, a comprehensive assessment of ARDS incidence and its associated risk factors in septic patients is urgently needed. This systematic review and meta-analysis aims to (1) quantify the pooled incidence of ARDS in patients with sepsis, and (2) identify key independent risk factors associated with its development, drawing from a wide range of observational studies across multiple healthcare settings.

## Materials and methods

### Study design and registration

This review was carried out following the methodological standards of the Cochrane Collaboration and is presented in line with the Preferred Reporting Items for Systematic Reviews and Meta-Analyses (PRISMA) guidelines. The study protocol was prospectively registered in the PROSPERO database (CRD420251118623).

### Data sources and searches

We carried out a comprehensive literature search across five major electronic databases: PubMed, Embase, the Cochrane Library, Web of Science, and Scopus. The search covered all publications available up to May 30, 2025. Both Medical Subject Headings (MeSH) and free-text terms were applied, using keywords such as sepsis, ARDS, incidence, risk factors, and predictors. To refine the strategy, Boolean operators (e.g., AND, odds ratios) were employed to combine terms effectively. Although no restrictions on language were set during the initial search, only studies published in English were considered for the final analysis. In addition, the reference lists of the included articles and relevant reviews were manually screened to capture any further eligible studies.

### Study selection

Titles and abstracts were first screened by two independent reviewers to determine relevance, after which the full texts of potentially eligible studies were assessed. Any disagreements during the selection process were addressed through discussion or, when necessary, consultation with a third reviewer. The inclusion of studies was guided by predefined eligibility criteria structured around the PICOS framework, which considers Population, Intervention/Exposure, Comparator, Outcomes, and Study design.

### Populations

Included studies enrolled adult patients (≥18 years) diagnosed with sepsis according to either the Sepsis-2 definitions (23) or Sepsis-3 definitions (5). Studies using the former Sepsis-1 criteria (24) were excluded to minimize diagnostic heterogeneity, as the SIRS-based framework is no longer recommended in current clinical practice.

### Interventions

As this was not a therapeutic meta-analysis, the term “intervention” refers to the observation and documentation of risk exposure or clinical characteristics in septic patients. These included clinical variables such as SOFA score, APACHE II score, infection site, comorbidities, and biochemical markers (e.g., CRP, lactate, etc.), which were hypothesized or tested as potential risk factors for ARDS development.

### Comparators

Comparators included patients with sepsis who did not develop ARDS. Studies that reported comparative data between ARDS and non-ARDS groups within septic populations were eligible. Both prospective and retrospective cohort designs were accepted.

### Outcomes

The primary outcome was the incidence of ARDS among patients with sepsis, as defined by the Berlin criteria (1). Studies that adopted older or nonspecified ARDS definitions (e.g., AECC 1994) were excluded to preserve diagnostic uniformity. Because the included studies rarely provided follow-up duration or person-time data, a time-based incidence rate could not be calculated. Therefore, our pooled estimate represents the percentage of sepsis patients experiencing ARDS during their hospital course. In this meta-analysis, the term “incidence” refers to the proportion of adult septic patients who developed ARDS during the observational or hospitalization period of each included study. Because most studies did not report follow-up time or person-years, the pooled estimate represents a cumulative incidence rather than a time-dependent incidence rate. Secondary outcomes included identification of statistically significant risk factors for ARDS in sepsis, expressed as OR, risk ratios, or mean differences with corresponding 95% confidence intervals (CI). For studies with multiple time points, data closest to the onset of ARDS were prioritized.

### Exclusion criteria

Studies were excluded if they (1) were not original research (e.g., reviews, editorials, conference abstracts); (2) lacked data on ARDS incidence or risk factors in sepsis; (3) included pediatric populations; (4) did not use standard definitions for sepsis and ARDS; (5) were case series, case reports, or non-comparative studies; or (6) had overlapping data with another included study (in which case, the most comprehensive or recent dataset was retained).

### Data extraction and management

Data were extracted independently by two reviewers using a standardized form. Extracted variables included: first author, year of publication, country, study design, sample size, diagnostic criteria, number of ARDS cases, incidence rate, and reported risk factors with effect sizes. For studies that provided graphical data only, we used digital extraction software (e.g., WebPlotDigitizer) to retrieve numerical values. Any discrepancies in data extraction were resolved through discussion.

### Assessment of risk of bias

The Newcastle-Ottawa Scale (NOS) was used to assess the quality of included observational studies (25). This scale evaluates studies across three domains: selection, comparability, and outcome (for cohort studies). Scores ≥7 were considered high quality. For randomized trials (if any), the Cochrane Risk of Bias Tool 2.0 was used. Two reviewers independently conducted quality assessments, with disagreements resolved by consensus.

### Data synthesis and analysis

Because most of the included studies reported comparative measures of association (such as odds ratios or mean differences) between ARDS and non-ARDS groups rather than stratified event proportions, the pooled analyses focused on these risk estimates. This approach allows the synthesis of both adjusted and unadjusted effects across heterogeneous study designs and avoids bias related to inconsistent thresholds (e.g., SOFA or APACHE II cut-offs) and sample weighting. As a result, the pooled results represent the relative strength of associations rather than absolute prevalence within each risk subgroup. All meta-analyses were carried out using Review Manager (version 5.4) and Stata (version 17). For incidence outcomes, pooled proportions were estimated with a random-effects model based on the DerSimonian and Laird approach. Risk factor analyses were summarized as pooled odds ratios (ORs) or mean differences (MDs) with corresponding 95% confidence intervals. Between-study heterogeneity was examined using the *I*^2^ statistic, with values greater than 50% considered indicative of substantial variation. In cases where marked heterogeneity was identified, subgroup analyses were performed according to study design, geographic region, and the definition of sepsis applied. Although variability across studies was anticipated, a meta-analysis was deemed appropriate because all selected studies addressed the same research question, applied comparable sepsis and ARDS definitions, and reported compatible outcome measures. A random-effects model was therefore used to account for heterogeneity, and subgroup and sensitivity analyses were performed to assess result stability. Effect sizes were pooled using either fixed-effects (labeled as ‘common’) or random-effects models according to heterogeneity. A fixed-effects model was applied when heterogeneity was low (*I*^2^ < 50% and *p* > 0.10), whereas a random-effects model (DerSimonian-Laird approach) was used when substantial heterogeneity was identified (*I*^2^ ≥ 50% or *p* ≤ 0.10). To assess potential publication bias, funnel plots and Egger’s test were employed when at least 10 studies contributed to a given outcome.

## Results

### Study selection

**Figure 1:**
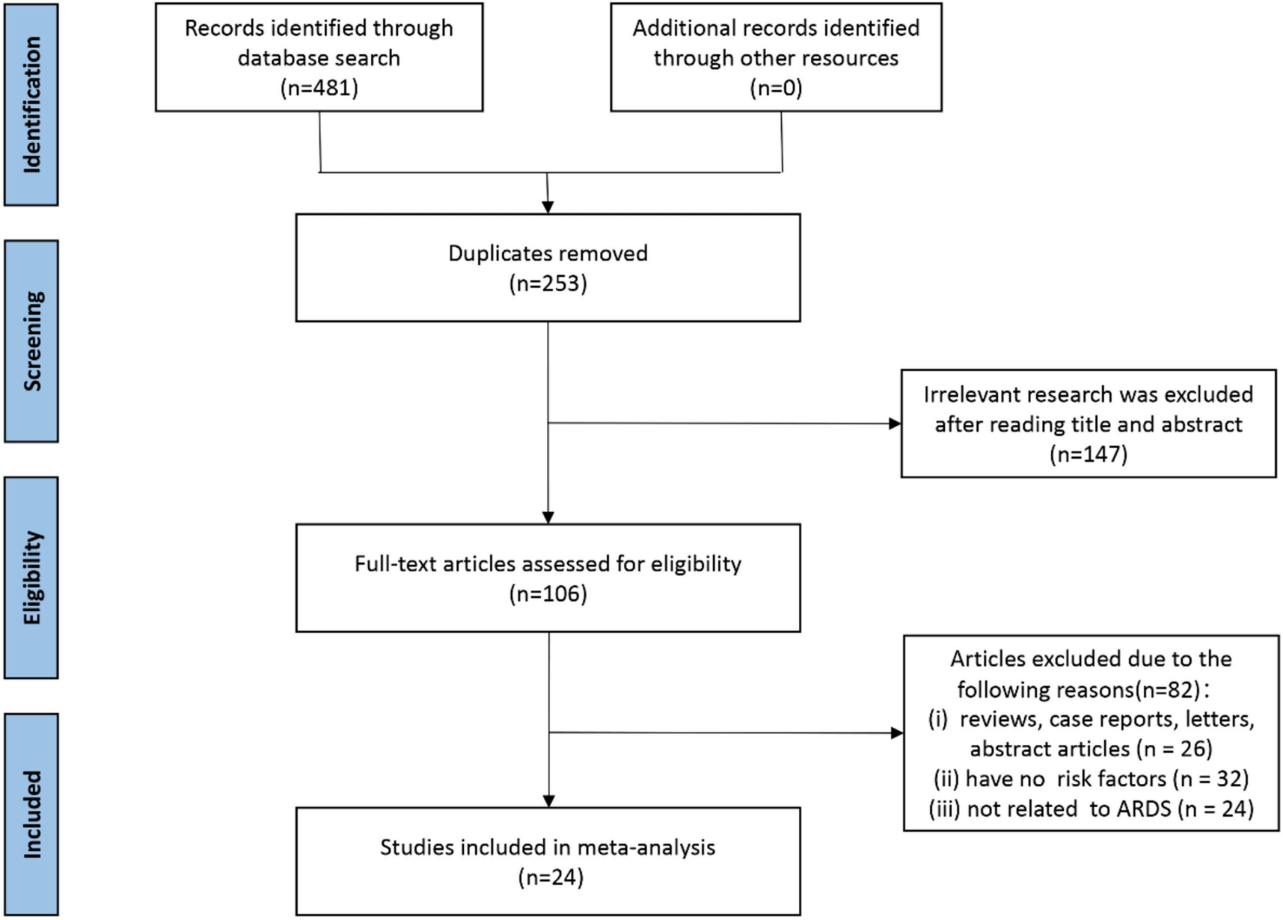
PRISMA flow diagram of study selection for inclusion in the meta-analysis.

A total of 481 records were initially identified through systematic database searches. No additional records were retrieved from manual searches or other sources. After removing 253 duplicates, 228 unique records remained for title and abstract screening. Subsequently, 147 records were excluded due to irrelevance to the study topic, leaving 106 full-text articles for eligibility assessment. Upon detailed evaluation, 82 studies were excluded for the following reasons: 26 were reviews, case reports, editorials, or abstracts without full-text access; 32 did not report any risk factors relevant to ARDS; and 24 were unrelated to ARDS despite involving sepsis populations. Ultimately, 24 studies (7, 10, 12–14, 26–43) met the inclusion criteria and were included in the final meta-analysis. The complete study selection process is illustrated in Figure 1.

### Study characteristics

A total of 24 studies were included in the analysis, comprising 19 prospective or retrospective cohort studies and one CCS. As summarized in Table 1, the studies were conducted across multiple countries, with the majority originating from China (*n* = 17), followed by the United States (*n* = 4), and one study each from Korea, Spain, and Japan. Sample sizes varied considerably, ranging from 50 to 11,566 participants. The proportion of male patients ranged from 48.0 to 73.4%. The majority of studies enrolled ICU patients, and only a small proportion involved mixed or undefined hospital populations, indicating that most data represented critically ill septic patients.

**Table 1:** Baseline characteristics of included studies reporting the prevalence of ARDS in patients with sepsis.

Author/year	Country	Study design	Sample size	Male (%)	Age, years	No. of ARDS in sepsis	Prevalence of ARDS in sepsis (%)
Xu 2023 (39)	China	RCS	11,566	6,959 (60.17)	65.45 ± 15.61	2,422	20.94
Auriemma 2020 (26)	US	PCS	811	424 (52.28)	–	294	36.25
Wang 2024 (35)	China	RCS	2,323	1,490 (64.1)	64 (50–77)	1,574	67.76
Li 2020 (29)	China	PCS	150	98 (65.3)	56.9 ± 10.3	41	27.33
Seethala 2017 (7)	US	PCS	2,534	1,298 (51.2)	58.5 ± 18.8	156	6.16
Wang 2020 (36)	China	RCS	322	195 (60.6)	62 (46–73)	84	26.09
Shi 2024 (33)	China	CCS	118	66 (55.93)	54.43 ± 10.07	35	29.66
Quan 2025 (32)	China	PCS	122	71 (58.20)	54.78 ± 10.06	32	26.23
Liu 2021	China	PCS	109	65 (59.6)	54.5 ± 10.7	28	25.69
Yang 2020 (42)	China	PCS	102	61 (59.8)	54.2 ± 10.9	26	25.49
Wang 2019 (37)	China	PCS	109	80 (73.4)	58.0 ± 10.7	32	29.36
Chen 2020 (28)	China	PCS	104	62 (59.6)	54.9 ± 10.9	30	28.85
Nam 2019 (14)	Korea	RCS	125	67 (53.6)	73 (59.0–80.0)	22	17.60
Bardají-Carrillo 2024 (27)	Spain	RCS	454	274 (60.35)	–	45	9.91
McKown 2017 (31)	US	RCS	1,080	594 (55.0)	57 (44–67)	441	40.83
Shi 2022 (12)	China	RCS	529	364 (68.8)	66 (53–76)	179	33.84
Ma 2024 (30)	China	RCS	738	485 (65.7)	66 (57–76)	218	29.54
Iriyama 2020 (10)	Japan	RCS	594	340 (57.24)	72 (62–81)	85	14.31
Yang 2022 (40)	US	PCS	111	67 (60.4)	65 (55–74)	21	18.92
Fan 2023	China	PCS	303	190 (62.7)	–	–	–
Mikkelsen 2013 (13)	US	RCS	778	421 (54.1)	55 (46–70)	48	6.17
Wu 2020 (38)	China	PCS	112	66 (58.9)	54.6 ± 11.0	30	26.79
Yang 2023 (41)	China	PCS	150	85 (56.66)	–	–	–
Sun 2024 (34)	China	RCS	50	24 (48)	69.28 ± 9.39	–	–

ARDS, acute respiratory distress syndrome; RCS, retrospective cohort study; PCS, prospective cohort study; CCS, case–control study.

The mean or median age of participants across the studies ranged from 54 to 73 years, though several studies did not report age distribution in detail. All studies employed the Berlin definition for ARDS diagnosis, which enhances comparability across datasets. Of the 24 studies, 21 reported the number of ARDS cases among patients with sepsis, with reported prevalence rates ranging from 6.16 to 67.76%. Notably, the highest prevalence was observed in a large retrospective cohort from China, while the lowest was reported in a prospective study from the United States. Three studies did not provide sufficient data to calculate ARDS prevalence and were thus excluded from quantitative synthesis.

### Incidence of ARDS among patients with sepsis

**Figure 2:**
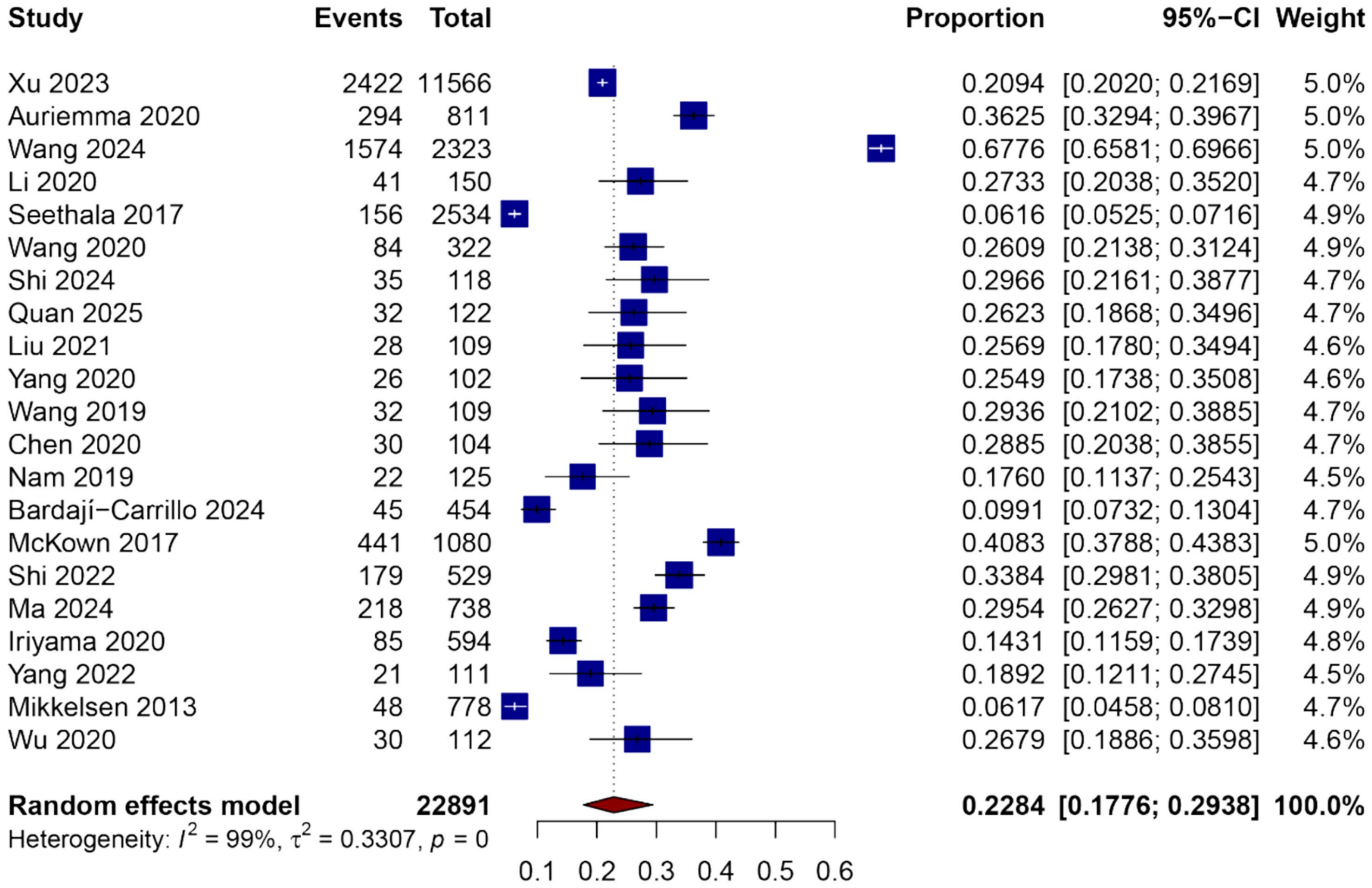
Forest plot of the pooled incidence of ARDS among patients with sepsis.

A total of 24 studies comprising 23,394 sepsis patients were included in the meta-analysis to estimate the pooled proportion of ARDS during hospitalization. The pooled incidence of ARDS among septic patients was 34.0% (95% CI: 29.0–39.3%), as shown in Figure 2. There was substantial heterogeneity across studies (*I*^2^ = 99%, *p* = 0), likely due to differences in study design, population characteristics, sepsis definitions, and diagnostic criteria for ARDS.

The reported incidence rates in individual studies ranged from 10.2 to 66.4%. Several studies conducted in ICU settings reported higher ARDS incidence compared to those in mixed or general ward populations. Subgroup analyses were planned to explore potential sources of heterogeneity, including geographic region, study design (prospective vs. retrospective), and sepsis definition criteria (Sepsis-2 vs. Sepsis-3), and are presented in subsequent sections.

Despite the observed heterogeneity, the overall effect estimate remained stable in sensitivity analyses, and no single study disproportionately influenced the pooled incidence. These results suggest that ARDS is a frequent complication among patients with sepsis and highlight the need for early identification and targeted management strategies in this vulnerable population.

### Assessment of publication bias

**Figure 3:**
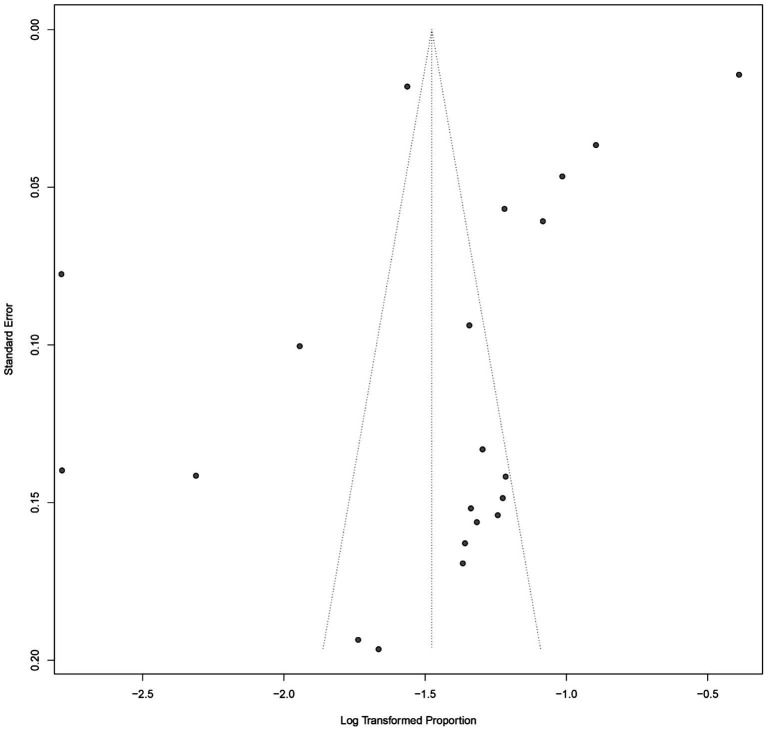
Funnel plot for assessing publication bias in the meta-analysis of ARDS incidence among patients with sepsis.

Publication bias was evaluated using a funnel plot of the studies reporting the incidence of ARDS in patients with sepsis (Figure 3). Visual inspection of the funnel plot revealed an asymmetrical distribution of studies, particularly with a relative absence of smaller studies reporting lower ARDS incidence. This pattern suggests the potential presence of publication bias or small-study effects.

To formally assess this, Egger’s regression test was performed and indicated statistically significant asymmetry (*p* = 0.012), further supporting the likelihood of publication bias. Begg’s rank correlation test also demonstrated marginal significance (*p* = 0.048). These findings suggest that studies with lower incidence estimates may be underrepresented in the published literature, potentially resulting in an overestimation of the pooled ARDS incidence. Sensitivity analyses using the trim-and-fill method were conducted to estimate the potential impact of publication bias on the summary estimate.

### Quality assessment of included studies

A total of 24 studies met the inclusion criteria for the final analysis, consisting of 23 cohort studies and one case–control investigation. The methodological quality of these studies was assessed using the Newcastle–Ottawa Scale (NOS), with summary results presented in Table 2 for cohort studies and Table 3 for the case–control study.

**Table 2:** Quality assessment of included cohort studies using the NOS.

Study	Selection				Comparability of cohorts	Outcomes			Score
	Representativeness of cohort	Selection of nonexposed cohort	Ascertainment of exposure	Outcome lacking at the beginning		Outcome assessment	Sufficient follow-up time	Follow up adequacy	
Xu 2023 (39)	★	★	★	★	★★	★	☆	★	8
Auriemma 2020 (26)	★	★	★	★	★★	★	★	★	9
Wang 2024 (35)	★	★	★	★	★★	★	☆	☆	7
Li 2020 (29)	★	★	★	★	★★	★	★	★	9
Seethala 2017 (7)	★	★	★	★	★★	★	★	★	9
Wang 2020 (36)	★	★	★	★	★★	★	☆	☆	7
Quan 2025 (32)	★	★	★	★	★★	☆	★	★	8
Liu 2021	★	★	★	★	★★	★	★	★	9
Yang 2020 (42)	★	★	★	★	★★	★	★	★	9
Wang 2019 (37)	★	☆	★	★	★☆	★	★	★	7
Chen 2020 (28)	★	★	★	★	★★	★	★	★	9
Nam 2019 (14)	★	★	★	★	★★	★	☆	★	8
Bardají-Carrillo 2024 (27)	★	☆	★	★	★☆	★	★	★	7
McKown 2017 (31)	★	☆	★	★	★☆	★	★	★	7
Shi 2022 (12)	★	☆	★	★	★☆	★	★	★	7
Ma 2024 (30)	★	☆	★	★	★☆	★	★	★	7
Iriyama 2020 (10)	★	☆	★	★	★☆	★	★	★	7
Yang 2022 (40)	★	☆	★	★	★☆	★	★	★	7
Fan 2023	★	☆	★	★	★☆	★	★	★	7
Mikkelsen 2013 (13)	★	☆	★	★	★☆	★	★	★	7
Wu 2020 (38)	★	★	★	★	★★	★	★	★	9
Yang 2023 (41)	★	☆	★	★	★☆	★	★	★	7
Sun 2024 (34)	★	☆	★	★	★☆	★	★	★	7

Notes: NOS, Newcastle–Ottawa Scale. ★ indicates that the study met the criterion and was awarded one star for the corresponding item; ☆ indicates that the criterion was not met and no star was awarded. In the Comparability domain, a study can receive up to two stars (★★).

**Table 3:** Quality assessment of included case–control studies using the NOS.

Study	Selection				Comparability	Exposure			Score
	Adequate definition of cases	Representativeness of the cases	Selection of controls	Definition of controls		Ascertainment of exposure	Same method of ascertainment for cases and controls	Non-response rate	
Shi 2024 (33)	★	★	★	★	★☆	★	★	★	8

Notes: NOS, Newcastle–Ottawa Scale. ★ indicates that the study met the criterion and was awarded one star for the corresponding item; ☆ indicates that the criterion was not met and no star was awarded. In the Comparability domain, a study can receive up to two stars (★★).

For the cohort studies, NOS scores ranged between 7 and 9, suggesting overall moderate to high quality. Notably, 11 studies (45.8%) obtained the maximum score of 9, demonstrating strong performance in key domains such as study selection, comparability of groups, and outcome assessment.

Most studies (*n* = 18, 75.0%) adequately reported follow-up periods and demonstrated low risk of attrition bias, although six studies were rated as having insufficient follow-up time or unclear follow-up adequacy. The majority also provided appropriate control for potential confounding variables, with 15 studies receiving two stars in the comparability domain.

The single included CCS (33) achieved a NOS score of 8 out of 9, reflecting high quality. It met all criteria in the selection and exposure domains and was awarded two stars for comparability. The study demonstrated rigorous case definition, appropriate control selection, and consistent ascertainment methods across groups.

Overall, the quality assessment suggests that the included studies were methodologically sound and suitable for meta-analysis. While minor limitations in follow-up reporting and comparability were observed in some studies, the overall risk of bias was low.

### Subgroup analysis of ARDS prevalence

To explore potential sources of heterogeneity, subgroup analyses were conducted based on study location and study design, as shown in Table 4. Geographical subgrouping revealed notable differences in ARDS prevalence among sepsis patients. In studies conducted in China (*n* = 13), the pooled prevalence was 29.62% (95% CI: 24.95–35.18), with substantial heterogeneity (*I*^2^ = 100%, *p* < 0.01). In contrast, studies from the United States (*n* = 5) reported a lower pooled prevalence of 16.10% (95% CI: 7.14–36.30), also with considerable heterogeneity (*I*^2^ = 99%, *p* < 0.01). Single studies from Korea, Spain, and Japan reported ARDS prevalence rates of 17.6, 9.91, and 14.31%, respectively, with relatively narrow confidence intervals despite the lack of pooled heterogeneity metrics. When stratified by study design, the pooled prevalence of ARDS in RCS (*n* = 10) was 21.92% (95% CI: 14.19–33.85; *I*^2^ = 100%, *p* < 0.01), while prospective cohort studies (PCS, *n* = 10) showed a comparable prevalence of 23.10% (95% CI: 16.75–31.85; *I*^2^ = 98%, *p* < 0.01). The single CCS yielded a prevalence of 29.66% (95% CI: 21.61–38.77), although this result should be interpreted cautiously due to the absence of pooled data. These findings suggest that both geographic region and study design may contribute to the observed heterogeneity in ARDS prevalence across studies. However, high between-study variability remained even within subgroups, indicating that additional factors such as patient characteristics, sepsis severity, and diagnostic practices may also play a role.

**Table 4:** Subgroup analysis of ARDS prevalence among sepsis patients by country and study design.

Subgroups	Number of included studies	Heterogeneity		Prevalence (%)	95%CI (%)	*p* value
		*I*^2^ (%)	*p* value			
Country						
China	13	100	<0.01	29.62	[24.95, 35.18]	<0.001
US	5	99	<0.01	16.10	[7.14, 36.30]	<0.001
Korea	1	–	–	17.6	[11.37, 25.43]	<0.001
Spain	1	–	–	9.91	[7.32, 13.04]	<0.001
Japan	1	–	–	14.31	[11.59, 17.39]	<0.001
Study design						
RCS	10	100	<0.01	21.92	[14.19, 33.85]	<0.001
PCS	10	98	<0.01	23.10	[16.75, 31.85]	<0.001
CCS	1	–	–	29.66	[21.61, 38.77]	<0.001

RCS, retrospective cohort study; PCS, prospective cohort study; CCS, case–control study.

### Meta-analysis of risk factors for ARDS in sepsis

A total of 25 potential risk factors for the development of ARDS in patients with sepsis were evaluated across the included studies (Table 5). Among these, several factors demonstrated statistically significant associations. Smoking was strongly associated with increased risk of ARDS, with a pooled odds ratio (OR) of 2.23 (95% CI: 1.33–3.75, *p* < 0.01; *I*^2^ = 72%), suggesting more than a twofold elevated risk among smokers. Similarly, respiratory infection (OR = 2.88, 95% CI: 2.07–3.99, *p* < 0.01; *I*^2^ = 83%) and (COPD) (OR = 3.09, 95% CI: 1.59–6.02, *p* < 0.01; *I*^2^ = 79%) were significantly associated with ARDS development. Septic shock also showed a positive correlation (OR = 1.78, 95% CI: 1.38–2.31, *p* < 0.01; *I*^2^ = 62%). Among laboratory parameters, elevated serum creatinine (Scr) (OR = 1.07, 95% CI: 1.05–1.10, *p* < 0.01; *I*^2^ = 0%), CRP (OR = 1.01, 95% CI: 1.01–1.01, *p* < 0.01; *I*^2^ = 85%), and white blood cell count (WBC) (OR = 1.02, 95% CI: 1.01–1.02, *p* < 0.01; *I*^2^ = 0%) were significantly associated with ARDS occurrence. In addition, higher APACHE II scores (OR = 1.12, 95% CI: 1.04–1.20, *p* < 0.01; *I*^2^ = 83%) and SOFA scores (OR = 1.15, 95% CI: 1.10–1.21, *p* < 0.01; *I*^2^ = 66%) were identified as significant predictors of ARDS in sepsis patients. Body mass index (BMI) also showed a modest but statistically significant effect (OR = 1.05, 95% CI: 1.01–1.08, *p* = 0.01; *I*^2^ = 70%). Conversely, several factors did not demonstrate significant associations with ARDS, including male gender (OR = 1.03, 95% CI: 0.95–1.11, *p* = 0.51), age (OR = 1.01, 95% CI: 0.99–1.03, *p* = 0.26), cirrhosis (OR = 0.80, *p* = 0.28), cardiomyopathy (OR = 1.19, *p* = 0.27), and diabetes (OR = 0.83, *p* = 0.25). Microbial etiologies such as Gram-negative, Gram-positive, and anaerobic/fungal/mycoplasmal organisms were also not significantly associated with increased ARDS risk. Although procalcitonin (PCT), IL-6, and TNF-α were analyzed in a limited number of studies, none showed a significant correlation with ARDS. Notably, pancreatitis was associated with a markedly increased risk (OR = 2.45, 95% CI: 1.87–3.21, *p* < 0.01; *I*^2^ = 0%), though this result was based on only three studies. These findings highlight several clinical and biochemical parameters that may serve as important indicators for ARDS risk stratification in septic patients, while also underscoring areas where evidence remains insufficient or conflicting.

**Table 5:** Meta-analysis of risk factors associated with the development of ARDS in patients with sepsis.

Risk factors	Number of included studies	Heterogeneity		Model	Overall effect	
		*I*^2^ (%)	*p* value		OR (95%CI)	*p* value
Male gender	14	28	0.16	Common	1.03 [0.95; 1.11]	0.51
Age	15	81	<0.01	Random	1.01 [0.99; 1.03]	0.26
BMI	12	70	<0.01	Random	1.05 [1.01; 1.08]	0.01
Smoking	11	72	<0.01	Random	2.23 [1.33; 3.75]	<0.01
Respiratory infection	12	83	<0.01	Random	2.88 [2.07; 3.99]	<0.01
COPD	9	79	<0.01	Random	3.09 [1.59; 6.02]	<0.01
Septic shock	6	62	0.02	Random	1.78 [1.38; 2.31]	<0.01
Cirrhosis	8	3	0.41	Common	0.80 [0.54; 1.20]	0.28
Cardiomyopathy	8	0	1.00	Common	1.19 [0.88; 1.61]	0.27
Chronic kidney failure	8	0	0.46	Common	1.42 [0.96; 2.11]	0.08
Diabetes	4	68	0.03	Random	0.83 [0.61; 1.13]	0.25
Pancreatitis	3	0	0.45	Common	2.45 [1.87; 3.21]	<0.01
CRP	10	85	<0.01	Random	1.01 [1.01; 1.01]	<0.01
Scr	9	0	0.69	Common	1.07 [1.05; 1.10]	<0.01
Albumin	8	0	0.96	Common	0.98 [0.96; 1.00]	0.13
PCT	5	89	<0.01	Random	1.63 [0.53; 5.07]	0.40
WBC	9	0	0.58	Common	1.02 [1.01; 1.02]	<0.01
IL-6	3	64	0.10	Random	0.63 [0.19; 2.06]	0.44
TNF-α	3	70	0.04	Random	0.99 [0.96; 1.02]	0.42
G− vs. others	5	0	0.96	Common	1.09 [0.73; 1.62]	0.68
G+ vs. others	6	0	0.88	Common	1.16 [0.78; 1.72]	0.47
Anaerobes/fungus/mycoplasmas vs. others	5	0	0.87	Common	1.16 [0.72; 1.87]	0.53
APACHE II score	15	83	<0.01	Random	1.12 [1.04; 1.20]	<0.01
SOFA score	12	66	<0.01	Random	1.15 [1.10; 1.21]	<0.01

BMI, body mass index; COPD, chronic obstructive pulmonary disease; CRP, C-reactive protein; Scr, serum creatinine; PCT, procalcitonin; WBC, white blood cell; IL-6, interleukin-6; TNF-α, tumor necrosis factor-α, G-, Gram-negative bacteria; G+, Gram-positive bacteria; PT, prothrombin time; APACHE II score, acute physiology and chronic health evaluation II score; SOFA score, Sequential Organ Failure Assessment score.

## Discussion

This systematic review and meta-analysis summarizes current evidence on the incidence and independent risk factors of ARDS among patients with sepsis. The pooled incidence was 34.0%, which aligns with earlier findings reported in both Western and Asian populations (3, 12). Nevertheless, substantial heterogeneity was observed across the included studies, likely due to differences in study design, patient characteristics, diagnostic definitions, and regional healthcare practices. Given the variability across study designs, populations, and reported prevalence rates, quantitative synthesis was essential to generate a consolidated estimate and to identify potential sources of heterogeneity among studies. It should be noted that, although the pooled outcome is expressed as “incidence,” the underlying data reflect the proportion of sepsis cases complicated by ARDS during hospitalization, as time-specific denominators were rarely reported. Therefore, the results should be interpreted as cumulative event proportions rather than true incidence rates. Future research should incorporate standardized follow-up times to enable more precise incidence estimation.

Our analysis identified several independent predictors of ARDS in sepsis, including elevated severity scores (SOFA and APACHE II), pulmonary infection, smoking, pancreatitis, septic shock, and elevated inflammatory or metabolic markers such as CRP and Scr. It is worth noting that the current analysis examined the relative association strength of risk factors using pooled odds ratios, not the absolute prevalence of ARDS among subgroups of septic patients with specific high-risk characteristics. This decision was made because individual studies rarely reported stratified incidence or prevalence by each risk factor. Pooling prevalence values directly would have introduced considerable uncertainty due to variable definitions and population heterogeneity. Therefore, the odds ratio-based meta-analytic approach provides a consistent and statistically robust means of estimating the comparative risks while acknowledging that absolute event rates by subgroup remain unavailable. These findings are in line with previous meta-analyses and cohort studies (7, 8, 10, 15, 19, 44), and reinforce the importance of comprehensive clinical assessment for risk stratification. Notably, the role of organ dysfunction scoring systems was strongly validated. Higher SOFA and APACHE II scores were consistently associated with increased ARDS risk across studies, with pooled OR demonstrating a stepwise increase in risk per point increment. Mayow et al. and Shi et al. (12) both confirmed the prognostic utility of these scores, especially SOFA, in predicting ARDS onset in ICU patients with sepsis (7, 8). The predictive strength of APACHE III, as shown by a machine learning model in Xu et al., further underscores the value of integrative scoring systems that incorporate physiological and biochemical parameters (44).

Pulmonary infections, especially pneumonia, were identified as a major and consistent contributor to both the onset and severity of ARDS. In a study by Shi et al., patients with pneumonia were found to have nearly a threefold greater risk of developing ARDS compared with those without pulmonary infection. Moreover, pneumonia was also linked to an increased likelihood of progression to severe ARDS, as defined by oxygenation thresholds in the Berlin criteria (7). These findings are consistent with earlier studies linking pulmonary sepsis to direct alveolar injury and heightened inflammatory responses (15, 16). Interestingly, Iriyama et al. highlighted that in non-pulmonary sepsis, soft tissue infections may also act as risk modifiers, suggesting that the infection site plays a variable role depending on the underlying etiology (10). Pancreatitis with local infection was another notable risk factor identified in both Shi et al. and the current meta-analysis. Its association with ARDS is likely due to systemic inflammatory responses leading to capillary leak and alveolar flooding, as previously described in experimental and clinical studies (45, 46). This finding supports the inclusion of pancreatitis in ARDS risk screening protocols for septic patients.

The role of smoking in ARDS development remains controversial. While our analysis found a significant association (OR = 2.23), some studies, including Iriyama et al., reported a paradoxically reduced risk in current smokers, possibly due to unmeasured confounders or selection bias (10). However, biomarker-based studies have demonstrated that cigarette smoke exposure induces epithelial injury and enhances profibrotic signaling, which supports its biological plausibility as a risk factor (47, 48). Laboratory markers such as CRP and Scr were also significantly associated with ARDS in sepsis. Elevated CRP reflects systemic inflammation and has been linked to worse outcomes in ARDS across multiple studies (49, 50). Similarly, elevated creatinine suggests renal dysfunction and multi-organ involvement, both of which are associated with increased lung injury risk.

The integration of machine learning approaches has shown considerable potential in predicting the risk and outcomes of ARDS. For instance, Xu et al. (44) reported that a random forest model achieved the strongest performance, with an AUROC of 0.846 for in-hospital mortality among patients with sepsis-related ARDS. In their analysis, bicarbonate, anion gap, and systolic blood pressure emerged as the most influential predictors (44). These variables were likewise identified in our analysis, further supporting their clinical importance. In contrast, several factors that have long been considered potential risks—including age, diabetes mellitus, cirrhosis, and chronic obstructive pulmonary disease—did not demonstrate significant associations with ARDS in the pooled results. This finding is consistent with prior studies (7, 8, 10), and supports the hypothesis that ARDS susceptibility is more strongly driven by acute disease severity and inflammatory burden than by chronic comorbidities. Nevertheless, this study has limitations. Another limitation is that nearly all included studies were based on ICU populations. Although this provides important information on sepsis-related ARDS among critically ill patients, it may limit the generalizability of the pooled prevalence to less severe or non-ICU sepsis cohorts. Future research should examine ARDS occurrence and risk factors in broader sepsis populations across different clinical settings. Substantial between-study heterogeneity may affect the generalizability of the pooled estimates. Although most studies used the Berlin definition for ARDS, inconsistencies in sepsis definitions (Sepsis-2 vs. Sepsis-3) may have influenced case classification. The majority of studies were retrospective and observational, which introduces inherent biases. Finally, publication bias was detected, suggesting that the true incidence of ARDS in sepsis may be lower than currently estimated. Future original studies should aim to present stratified data—such as ARDS prevalence among septic patients with elevated SOFA or APACHE II scores or septic shock—to facilitate more clinically interpretable risk quantification.

## Conclusion

This meta-analysis reveals that ARDS develops in approximately one-third of patients with sepsis and is primarily driven by acute illness severity rather than baseline comorbidities. Pneumonia, pancreatitis with local infection, septic shock, elevated SOFA and APACHE II scores, and inflammatory markers such as CRP and creatinine emerged as key risk factors. These findings underscore the need for early risk identification and support the integration of clinical and biochemical indicators into routine assessment to guide timely intervention and improve outcomes in septic patients.

## Data Availability

The datasets presented in this study can be found in online repositories. The names of the repository/repositories and accession number(s) can be found in the article/supplementary material.

## References

[1] Ranieri VM Rubenfeld GD Thompson BT Ferguson ND Caldwell E Fan E . Acute respiratory distress syndrome: the Berlin definition. JAMA. (2012) 307:2526–33. doi: 10.1001/jama.2012.5669, 22797452

[2] Matthay MA Ware LB Zimmerman GA. The acute respiratory distress syndrome. J Clin Invest. (2012) 122:2731–40. doi: 10.1172/JCI60331, 22850883 PMC3408735

[3] Bellani G Laffey JG Pham T Fan E Brochard L Esteban A . Epidemiology, patterns of care, and mortality for patients with acute respiratory distress syndrome in intensive care units in 50 countries. JAMA. (2016) 315:788–800. doi: 10.1001/jama.2016.0291, 26903337

[4] Villar J Blanco J Añón JM Santos-Bouza A Blanch L Ambrós A . The ALIEN study: incidence and outcome of acute respiratory distress syndrome in the era of lung protective ventilation. Intensive Care Med. (2011) 37:1932–41. doi: 10.1007/s00134-011-2380-4, 21997128

[5] Singer M Deutschman CS Seymour CW Shankar-Hari M Annane D Bauer M . The third international consensus definitions for sepsis and septic shock (Sepsis-3). JAMA. (2016) 315:801–10. doi: 10.1001/jama.2016.0287, 26903338 PMC4968574

[6] Rudd KE Johnson SC Agesa KM Shackelford KA Tsoi D Kievlan DR . Global, regional, and national sepsis incidence and mortality, 1990-2017: analysis for the global burden of disease study. Lancet. (2020) 395:200–11. doi: 10.1016/S0140-6736(19)32989-7, 31954465 PMC6970225

[7] Seethala RR Hou PC Aisiku IP Frendl G Park PK Mikkelsen ME . Early risk factors and the role of fluid administration in developing acute respiratory distress syndrome in septic patients. Ann Intensive Care. (2017) 7:11. doi: 10.1186/s13613-017-0233-1, 28116595 PMC5256622

[8] Mayow AH Ahmad F Afzal MS Khokhar MU Rafique D Vallamchetla SK . A systematic review and meta-analysis of independent predictors for acute respiratory distress syndrome in patients presenting with sepsis. Cureus. (2023) 15:e37055. doi: 10.7759/cureus.37055, 37143620 PMC10153762

[9] Yin R Yang X Yao Y. Risk factors for acute respiratory distress syndrome in sepsis patients: a meta-analysis. Heliyon. (2024) 10:e37336. doi: 10.1016/j.heliyon.2024.e37336, 39309902 PMC11414502

[10] Iriyama H Abe T Kushimoto S Fujishima S Ogura H Shiraishi A . Risk modifiers of acute respiratory distress syndrome in patients with non-pulmonary sepsis: a retrospective analysis of the FORECAST study. J Intensive Care. (2020) 8:7. doi: 10.1186/s40560-020-0426-9, 31938547 PMC6954566

[11] Gong MN Thompson BT Williams P Pothier L Boyce PD Christiani DC. Clinical predictors of and mortality in acute respiratory distress syndrome: potential role of red cell transfusion. Crit Care Med. (2005) 33:1191–8. doi: 10.1097/01.CCM.0000165566.82925.14, 15942330

[12] Shi Y Wang L Yu S Ma X Li X. Risk factors for acute respiratory distress syndrome in sepsis patients: a retrospective study from a tertiary hospital in China. BMC Pulm Med. (2022) 22:238. doi: 10.1186/s12890-022-02015-w, 35729588 PMC9210689

[13] Mikkelsen ME Shah CV Meyer NJ Gaieski DF Lyon S Miltiades AN . The epidemiology of acute respiratory distress syndrome in patients presenting to the emergency department with severe sepsis. Shock. (2013) 40:375–81. doi: 10.1097/SHK.0b013e3182a64682, 23903852 PMC3800497

[14] Nam H Jang SH Hwang YI Kim JH Park JY Park S. Nonpulmonary risk factors of acute respiratory distress syndrome in patients with septic bacteraemia. Korean J Intern Med. (2019) 34:116–24. doi: 10.3904/kjim.2017.204, 29898577 PMC6325442

[15] Sheu CC Gong MN Zhai R Chen F Bajwa EK Clardy PF . Clinical characteristics and outcomes of sepsis-related vs non-sepsis-related ARDS. Chest. (2010) 138:559–67. doi: 10.1378/chest.09-2933, 20507948 PMC2940067

[16] Fujishima S Gando S Daizoh S Kushimoto S Ogura H Mayumi T . Infection site is predictive of outcome in acute lung injury associated with severe sepsis and septic shock. Respirology. (2016) 21:898–904. doi: 10.1111/resp.12769, 27028604

[17] Trillo-Alvarez C Cartin-Ceba R Kor DJ Kojicic M Kashyap R Thakur S . Acute lung injury prediction score: derivation and validation in a population-based sample. Eur Respir J. (2011) 37:604–9. doi: 10.1183/09031936.00036810, 20562130

[18] Moss M Guidot DM Steinberg KP Duhon GF Treece P Wolken R . Diabetic patients have a decreased incidence of acute respiratory distress syndrome. Crit Care Med. (2000) 28:2187–92. doi: 10.1097/00003246-200007000-00001, 10921539

[19] Calfee CS Matthay MA Kangelaris KN Siew ED Janz DR Bernard GR . Cigarette smoke exposure and the acute respiratory distress syndrome. Crit Care Med. (2015) 43:1790–7. doi: 10.1097/CCM.0000000000001089, 26010690 PMC4737582

[20] Shyamsundar M McKeown ST O'Kane CM Craig TR Brown V Thickett DR . Simvastatin decreases lipopolysaccharide-induced pulmonary inflammation in healthy volunteers. Am J Respir Crit Care Med. (2009) 179:1107–14. doi: 10.1164/rccm.200810-1584OC, 19324974 PMC2695496

[21] Peter JV John P Graham PL Moran JL George IA Bersten A. Corticosteroids in the prevention and treatment of acute respiratory distress syndrome (ARDS) in adults: meta-analysis. BMJ. (2008) 336:1006–9. doi: 10.1136/bmj.39537.939039.BE, 18434379 PMC2364864

[22] Levitt JE Matthay MA. Clinical review: early treatment of acute lung injury--paradigm shift toward prevention and treatment prior to respiratory failure. Crit Care. (2012) 16:223. doi: 10.1186/cc11144, 22713281 PMC3580596

[23] Levy MM Fink MP Marshall JC Abraham E Angus D Cook D . 2001 SCCM/ESICM/ACCP/ATS/SIS international sepsis definitions conference. Crit Care Med. (2003) 31:1250–6. doi: 10.1097/01.CCM.0000050454.01978.3B, 12682500

[24] Bone RC Balk RA Cerra FB Dellinger RP Fein AM Knaus WA . Definitions for sepsis and organ failure and guidelines for the use of innovative therapies in sepsis. The ACCP/SCCM Consensus Conference Committee. American College of Chest Physicians/Society of Critical Care Medicine. Chest. (1992) 101:1644–55. doi: 10.1378/chest.101.6.1644, 1303622

[25] Stang A. Critical evaluation of the Newcastle-Ottawa scale for the assessment of the quality of nonrandomized studies in meta-analyses. Eur J Epidemiol. (2010) 25:603–5. doi: 10.1007/s10654-010-9491-z, 20652370

[26] Auriemma CL Zhuo H Delucchi K Deiss T Liu T Jauregui A . Acute respiratory distress syndrome-attributable mortality in critically ill patients with sepsis. Intensive Care Med. (2020) 46:1222–31. doi: 10.1007/s00134-020-06010-9, 32206845 PMC7224051

[27] Bardají-Carrillo M Martín-Fernández M López-Herrero R Priede-Vimbela JM Heredia-Rodríguez M Gómez-Sánchez E . Post-operative sepsis-induced acute respiratory distress syndrome: risk factors for a life-threatening complication. Front Med (Lausanne). (2024) 11:1338542. doi: 10.3389/fmed.2024.1338542, 38504911 PMC10948508

[28] Chen W Liu L Yang J Wang Y. MicroRNA-146b correlates with decreased acute respiratory distress syndrome risk, reduced disease severity, and lower 28-day mortality in sepsis patients. J Clin Lab Anal. (2020) 34. doi: 10.1002/jcla.23510, 32845540 PMC7755760

[29] Li S Zhao D Cui J Wang L Ma X Li Y. Prevalence, potential risk factors and mortality rates of acute respiratory distress syndrome in Chinese patients with sepsis. J Int Med Res. (2020) 48. doi: 10.1177/0300060519895659, 32043378 PMC7105739

[30] Ma Y Zhu C Ma X Zhou B Dong M. Risk factors of acute respiratory distress syndrome in sepsis caused by intra-abdominal infections: a retrospective study. Surgery. (2024) 175:1432–8. doi: 10.1016/j.surg.2024.01.020, 38383244

[31] McKown AC McGuinn EM Ware LB Wang L Janz DR Rice TW . Preadmission oral corticosteroids are associated with reduced risk of acute respiratory distress syndrome in critically ill adults with sepsis*. Crit Care Med. (2017) 45:774–80. doi: 10.1097/CCM.0000000000002286, 28257336 PMC5392158

[32] Quan Y Gao S. LncRNA HOXA-AS2 can predict the risk of acute respiratory distress syndrome and 28-day mortality in patients with sepsis. Clin Respir J. (2025) 19. doi: 10.1111/crj.70082, 40396530 PMC12093249

[33] Shi W Zhu W Yu J Shi Y Zhao Y. LncRNA HOTTIP as a diagnostic biomarker for acute respiratory distress syndrome in patients with sepsis and to predict the short-term clinical outcome: a case-control study. BMC Anesthesiol. (2024) 24. doi: 10.1186/s12871-024-02405-z, 38238652 PMC10795278

[34] Sun C Xie Y Zhu C Guo L Wei J Xu B . Serum Mrp 8/14 as a potential biomarker for predicting the occurrence of acute respiratory distress syndrome induced by sepsis: a retrospective controlled study. J Inflamm Res. (2024) 17:2939–49. doi: 10.2147/JIR.S457547, 38764498 PMC11100500

[35] Wang D-H Jia H-M Zheng X Xi X-M Zheng Y Li W-X. Attributable mortality of ARDS among critically ill patients with sepsis: a multicenter, retrospective cohort study. BMC Pulm Med. (2024) 24. doi: 10.1186/s12890-024-02913-1, 38438849 PMC10913263

[36] Wang Y-m. Effects of fluid balance on prognosis of acute respiratory distress syndrome patients secondary to sepsis. World J Emerg Med. (2020) 11. doi: 10.5847/wjem.j.1920-8642.2020.04.003, 33014217 PMC7517398

[37] Wang Y Fu X Yu B Ai F. Long non-coding RNA THRIL predicts increased acute respiratory distress syndrome risk and positively correlates with disease severity, inflammation, and mortality in sepsis patients. J Clin Lab Anal. (2019) 33. doi: 10.1002/jcla.22882, 31257645 PMC6642293

[38] Wu X Chen D Yu L. The value of circulating long non-coding RNA maternally expressed gene 3 as a predictor of higher acute respiratory distress syndrome risk and 28-day mortality in sepsis patients. J Clin Lab Anal. (2020) 34. doi: 10.1002/jcla.23488, 32844492 PMC7676220

[39] Xu C Zheng L Jiang Y Jin L. A prediction model for predicting the risk of acute respiratory distress syndrome in sepsis patients: a retrospective cohort study. BMC Pulm Med. (2023) 23. doi: 10.1186/s12890-023-02365-z, 36890503 PMC9994387

[40] Yang P Iffrig E Harris F Holder AL Martin GS Esper AM. Serial measurements of protein biomarkers in sepsis-induced acute respiratory distress syndrome. Crit Care Explor. (2022) 4. doi: 10.1097/CCE.0000000000000780, 36284549 PMC9586925

[41] Yang Q Zhang X Luo L Shen J. Clinical application of serum NLRP3 on the diagnosis and prognosis of sepsis patients complicated with acute respiratory distress syndrome. Front Immunol. (2023) 14. doi: 10.3389/fimmu.2023.1205132, 37649483 PMC10462769

[42] Yang Y Yang L Liu Z Wang Y Yang J. Long noncoding RNA NEAT 1 and its target microRNA-125a in sepsis: correlation with acute respiratory distress syndrome risk, biochemical indexes, disease severity, and 28-day mortality. J Clin Lab Anal. (2020) 34. doi: 10.1002/jcla.23509 PMC7755762 32785981

[43] Yijue L Huan P Feng G. Long noncoding plasmacytoma variant translocation 1 facilitates the surveillance of acute respiratory distress syndrome and mortality prediction in sepsis. Biomark Med. (2021) 15. doi: 10.2217/bmm-2020-0506 33733809

[44] Xu Z Zhang K Liu D Fang X. Predicting mortality and risk factors of sepsis related ARDS using machine learning models. Sci Rep. (2025) 15:13509. doi: 10.1038/s41598-025-96501-w, 40251182 PMC12008361

[45] Shields CJ Winter DC Redmond HP. Lung injury in acute pancreatitis: mechanisms, prevention, and therapy. Curr Opin Crit Care. (2002) 8:158–63. doi: 10.1097/00075198-200204000-00012, 12386518

[46] Raghu MG Wig JD Kochhar R Gupta D Gupta R Yadav TD . Lung complications in acute pancreatitis. JOP. (2007) 8:177–85. 17356240

[47] Checa M Hagood JS Velazquez-Cruz R Ruiz V García-De-Alba C Rangel-Escareño C . Cigarette smoke enhances the expression of profibrotic molecules in alveolar epithelial cells. PLoS One. (2016) 11:e0150383. doi: 10.1371/journal.pone.0150383, 26934369 PMC4775036

[48] Hsieh SJ Ware LB Eisner MD Yu L Jacob P 3rd Havel C . Biomarkers increase detection of active smoking and secondhand smoke exposure in critically ill patients. Crit Care Med. (2011) 39:40–5. doi: 10.1097/CCM.0b013e3181fa4196, 20935560 PMC3148017

[49] Spadaro S Park M Turrini C Tunstall T Thwaites R Mauri T . Biomarkers for acute respiratory distress syndrome and prospects for personalised medicine. J Inflamm Lond. (2019) 16:1. doi: 10.1186/s12950-018-0202-y 30675131 PMC6332898

[50] Ware LB Koyama T Zhao Z Janz DR Wickersham N Bernard GR . Biomarkers of lung epithelial injury and inflammation distinguish severe sepsis patients with acute respiratory distress syndrome. Crit Care. (2013) 17:R253. doi: 10.1186/cc13080, 24156650 PMC4056313

